# The Surgical Treatment of Bronchiectasis
*Report of a paper given before the Devon and Exeter Medico-Chirurgical Society on 3rd April, 1952.


**Published:** 1952-07

**Authors:** J. L. Griffith

**Affiliations:** Thoracic Surgeon, Devon and Cornwall Area


### THE SURGICAL TREATMENT OF BRONCHIECTASIS*

BY

J. L. GRIFFITH, F.R.C.S.

*Thoracic Surgeon, Devon and Cornwall Area*

Bronchiectasis is a permanent and pathological dilatation of a bronchus altering its function. It can only be cured by removal of the diseased areas. Medical treatment is palliative and consists of change of environment to a more congenial climate, and control of respiratory infections by physiotherapy and antibiotics when necessary. Once established the condition tends to get worse with repeated infections, the result of " spill over " of sputum from infected areas of the lung to the healthy areas. Common complications are cerebral abscess and focal nephritis. Nasal sepsis may be a primary cause of the bronchial infection, or a secondary chronic sinusitis may arise later. That nasal secretions reach the chest during sleep can be proved by instilling lipiodol into the nose, when some of it can be shown on X-ray to be in the chest on the following day. Again, excision of a bronchiectatic area quite often clears a chronic nasal sepsis. Before the use of antibiotics few patients with much disease survived over fifty years. The patient with bronchiectasis who needs surgical treatment sometimes fails to get it because of the priority given to tuberculous patients waiting for operation.

Two forms occur, the wet and the dry. In the wet form the patient has mucoid or purulent sputum; in the mild case there is sputum in the morning only, in the moderate case sputum in the morning all the year round, generally with a history of repeated bronchitis or pneumonia. In the severe case there is copious sputum with dyspnoea and persistent cough. The mild and moderate types are suitable for treatment, but the severe cases are usually not amenable to surgical treatment. In the wet form of the disease, the lower lobes are usually involved. There is a middle lobe type, the Brock syndrome, the basic cause being epituberculosis. In the dry form there is little cough and no sputum, but there are bouts of haemoptysis. The upper lobes are affected. The sputum is scanty, because there is good drainage in the upright posture. Surgical treatment is not advisable for these cases unless haemoptyses are frequent. When operation is done the bronchial arteries are found to be dilated as well as the bronchi. Large doses of hypnotics should not be given for haemoptyses in these cases, owing to the risk of atelectasis from blood clot.

The physical signs are clubbing of the fingers and foetor in advanced disease, but more useful is the early " laughing sign " (the patient usually coughs on laughing). The exercise sign is very suggestive of bronchiectasis, and the patient who " likes a cigarette first thing in the morning to get rid of the sputum"

* Report of a paper given before the Devon and Exeter Medico-Chirurgical Society on 3rd April, 1952.

is also probably a sufferer. Rales will be detected over the diseased area on clinical examination. The diagnosis can usually be made on straight X-ray films, particularly a lateral view. In children, bronchograms are often essential. The X-ray may show bunching of the root shadows and collapsed segments. A bronchogram will show either a saccular appearance or finger-like dilatation of the affected bronchi. Sputum examination for tubercle bacilli and fungi should be undertaken. Bronchoscopy is performed to make sure that the bronchi are patent. Before the bronchoscopy the chest must be cleared of sputum or it may be difficult to make a satisfactory examination.

### MEDICAL TREATMENT

Cases over forty to forty-five years are usually too old for operation because of associated suppurative bronchitis, and toxic nephritis is a contra-indication to operation. The lower age limit is determined by the age the particular patient can co-operate with the physiotherapist, usually about six to eight years. Asthma and emphysema are contra-indications to surgery as a spasm of asthma after operation may lead to atelectasis.

### SURGICAL TREATMENT

In 1911, ligature of the pulmonary artery, thoracoplasty, artificial pneumothorax and phrenicectomy were all tried. Later mass ligation of the hilum was done, usually with bad results. In 1928, tourniquet lobectomy was developed, but bronchial fistula resulted in 30 per cent. of the cases, and the long bronchial stump remaining acted as a reservoir of infection. Churchill and Belsey (1939) introduced segmental lobectomy, making a great advance. The upper lobe of the lung is composed of three segments, the middle lobe two and the lower lobe four or five, and it is possible to peel off a diseased segment from a healthy lung in the same way that a gall bladder is peeled off from the under surface of the liver in cholecystectomy. Thus it is possible, by removing diseased segments only, to conserve much of the healthy lung which would otherwise be sacrificed, and bilateral operations can be performed with greater safety.

Pre-operative treatment takes three to six weeks for the cleaning up of bronchial secretion—to prevent atelectasis. Intensive breathing exercises (diaphragmatic, costal and shoulder girdle exercises) are instituted. By this means the vital capacity can often be increased by as much as 500 cc. Postural drainage is done three to four times a day, and the physiotherapist aims to empty each lobe in turn, and charts the quantity obtained each day.

Nasal sepsis is attended to and intrabronchial installation of penicillin into diseased lobes is performed daily. Expectorant mixtures are of little value and smoking is prohibited. Anti-spasmodics are helpful and anaemia is corrected. When the sputum is minimal bronchoscopy is done to look for any obstruction; this is best carried out under local anaesthesia so that the patient's co-operation can be obtained and voluntary coughing is not abolished. Bronchography is also best done when the sputum is minimal and the lipiodol must be emptied out of the lung afterwards to avoid post-operative atelectasis. A recent innovation for this is *Ioduron B*, a water-soluble dye, which is rapidly absorbed and excreted from the lungs.

### REFERENCE

Churchill, E. D., and Belsey, R. (1939). *Ann. Surg.* **109**, 481.

